# Early identification of major depression risk among reproductive-age women using hormonal contraceptives in southern Ethiopia: A Machine Learning Approach

**DOI:** 10.1038/s41598-026-54084-0

**Published:** 2026-05-25

**Authors:** Dagne Deresa Dinagde, Adugna Alemu Desta, Esayas Tadele, Dagim Tsegaye, Habtamu Wana Wada

**Affiliations:** 1 Department of Midwifery, College of Medicine and Health Sciences, Salale University, Fitche, Ethiopia; 2 Department of Public Health, College of Health Sciences, Mattu University, Mattu, Ethiopia; 3 Department of Biomedical Science, College of Health Sciences, Mattu University, Mattu, Ethiopia; 4 Department of Midwifery, Arba Minch College of Health Sciences, Arba Minch, Ethiopia

**Keywords:** Depression, Hormonal contraception, Mental health, Machine learning, Ethiopia, Computational biology and bioinformatics, Diseases, Health care, Mathematics and computing, Medical research, Risk factors

## Abstract

Machine-learning (ML) algorithms are increasingly valuable in health sciences because they can analyze complex, high-dimensional data and detect patterns that may not be easily identified using traditional statistical methods. These models can efficiently classify individuals into low- and high-risk groups, enabling early detection of conditions such as depression, cancer, and diabetes before symptoms become severe. Thus, by revealing intricate patterns in sizable health datasets, machine-learning analysis facilitates the precise and early identification of high-risk patients, facilitating prompt diagnosis, efficient screening, and well-informed clinical decision-making. Therefore, the aim of this study is to develop a predictive model for major depression in women using hormonal contraceptives in southern Ethiopia. A cross-sectional data was collected from 1002 participants over two consecutive months from eight public hospitals in Gamo Zone southern Ethiopia from August 1 to September 30, 2025. Six machine-learning models, random forest, decision tree, logistic regression, partial decision tree (PART), Naïve Bayes, and XGBoost, were employed to predict the probability of developing major depression. The dataset was split into a training set (80% of the observations) and a test set (20% of the observations). The predictive capacities of each machine-learning model were evaluated using receiver operating characteristic curves and various measures of model performance. The SHAP and Gini information values were used in selection of important attributes for predicting major depression. The predictive performance of the six machine-learning models demonstrated Cohen’s Kappa values ranging from 0.185 to 0.480, indicating slight to moderate agreement beyond chance. Among the algorithms tested, the random forest model demonstrated the highest accuracy (84.4%) and precision (93.2%). Using fivefold cross-validation and SMOTE to address data imbalance, the model achieved a high F1-score (91%) and the highest AUC (0.831) on the test data, indicating good discriminatory power. Feature-importance analysis using SHAP values identified maternal occupation and younger maternal age as the key attributes associated with an increased probability of major depression among hormonal contraceptive users, whereas longer duration of method use was identified as a protective factor associated with a 16% lower risk. The Random Forest model demonstrated the best predictive performance for major depression, achieving comparatively higher accuracy, recall, precision, F1-score and AUC values than the other models evaluated. Therefore, clinicians and healthcare researchers consider using the Random Forest model as a supportive decision-making tool for early risk prediction and targeted intervention.

## Background

Depression is a typical mental disorder that manifests as a range of emotional, physical, and behavioral symptoms, including difficulty concentrating, feelings of overwhelming guilt or low self-worth, suicidal thoughts, disturbed sleep, and decreased energy for at least 14 days^1^. Depression is the fourth leading cause of disability worldwide and is expected to overtake all other major causes of disability in the near future^2^. Depression can have serious consequences for women and their families. It is often associated with higher rates of anxiety, social isolation, substance abuse, and disruptions in relationships with children and partners^3^. Thus, Hormonal changes are thought to contribute to the development of depression. Specific alterations in brain functioning can lead to hormonal imbalances, which in turn play a role in the emergence of depressive symptoms^4^.

Standard cut-off scores on validated instruments can be used to classify the severity of major depression. A score of ≥ 10 is frequently used as a threshold for clinically significant major depressive disorder on the Patient Health Questionnaire-9 (PHQ-9), which has a range of 0–27^5^. On the Beck Depression Inventory (BDI-II), which has a range of 0–63, a score of 0–13 indicates minor depression, 14–19 mild depression, 20–28 moderate depression, and 29–63 severe depression^6^. Scores of 0–7 are regarded as normal, 8–13 as mild, 14–18 as moderate, 19–22 as severe, and ≥ 23 as extremely severe depression on the clinician-rated Hamilton Depression Rating Scale (HAM-D, 17-item version)^7^. Instead of being the exclusive foundation for diagnosis, these cut-offs aid in determining severity and directing clinical decision-making.

More than 264 million people worldwide are affected by depression. At least 25% of all women and 15% of all men will experience a depressive episode that requires treatment at some point in their lifetime^8^. According to the WHO’s Global Burden of Disease Study, major depression is estimated to affect about 8% to 16% of reproductive-age women in the world^9^. Globally, about 9.4% of women who have been using hormonal contraception will develop depression symptoms^10^. A large Danish registry study involving over 1 million women found that during the initial months of using combined oral contraceptives (COCs), the risk of receiving a first diagnosis of depression or initiating antidepressant use increased by approximately 1.8 times. Additionally, the study estimated that the risk of suicide or suicide attempts increased by around twofold during this early period of COC usage^11^.

Computer systems may learn from data and prior experiences, recognize patterns, and gradually enhance their performance without explicit programming thanks to machine learning (ML), a subfield of artificial intelligence^12^. It does this by optimizing model parameters (i.e. internal variables) through calculations, such that the model’s behavior reflects the data or experience. The learning algorithm then continuously updates the parameter values as learning progresses, enabling the ML model to learn and make predictions or decisions based on data science^13^. There are two primary categories into which data mining tasks can be divided. Predictive data mining uses supervised and unsupervised machine learning techniques for inference in order to make predictions based on the insights obtained from the data, whereas descriptive data mining seeks to describe the general characteristics of a specific dataset^14–16^.

The utilization of machine-learning (ML) is progressively growing within the medical sector. In recent years, supervised machine learning approaches have been increasingly applied in field of health science including mental health research to predict major depression at the population level^17^. Using labeled datasets, where people are categorized based on established screening instruments or standardized diagnostic criteria, these techniques enable algorithms to identify patterns linked to depressed symptoms. Depression status is the outcome variable, while sociodemographic, clinical, behavioral, and psychosocial aspects are included as predictor variables. Common supervised algorithms, including logistic regression, decision trees, random forest, support vector machines (SVM), Naïve Bayes, k-nearest neighbors (KNN), and gradient boosting techniques such as XGBoost, have demonstrated promising performance in identifying high-risk individuals. Such data-driven approaches enhance early detection, risk stratification, and informed decision-making in public mental health practice.

To the researchers’ knowledge, only a few ordinary studies have conducted in Ethiopia to examine the association between hormonal contraception and mental health issues like anxiety and depression and all previous studies mainly focused on numerical association of certain related factors. The best machine learning algorithms for predicting major depression in women of reproductive age that use hormonal contraception have not yet been investigated in any Ethiopian or African studies. There is still little data linking advanced predictive analytics to reproductive health, despite the region’s rising prevalence of mental health issues and rising use of contraceptive techniques. Thus, the long run aim of this study is early identification of major depression risk among hormonal contraceptive users in south Ethiopia using machine-learning algorithms.

## Methods and materials

### Study design and setting

The study was conducted in public health facilities in Southern Ethiopia’s Gamo Zone. Arba Minch Town serves as the zone’s administrative hub. It is located roughly 275 km southwest of Hawassa and 495 km from Ethiopia’s capital, Addis Ababa. According to the Central Statistical Agency of Ethiopia’s (CSA) 2007 national census, there are 1,659,310 people living in the Gamo Zone, with 779,332 males and 879,782 women^16,18^. Regarding healthcare infrastructure, the Gamo Zone has a total of 63 public health facilities, including 57 health centers and 8 hospitals. All hospitals, with the exception of Arba Minch Comprehensive and Teaching Hospital, provide a range of services including inpatient and outpatient medical care, surgical procedures, antenatal care, intrapartum and postnatal care, immunization, pediatric services, and family planning. This study was carried out over a two-month period, from August 1 to September 30, 2025 within these public hospitals.

### Data sources and study population

Quantitative data were collected from all reproductive-age women receiving hormonal contraception at public hospitals across the Gamo Zone over a consecutive two-month period, from August 1 to September 30, 2025. The target population for this study comprised all women of reproductive age (15–49 years) who were using hormonal contraceptive methods and seeking health services at the selected hospitals in the Gamo zone, southern Ethiopia. Women who visited the selected hospitals during the data collection period and had been consistently utilizing hormonal contraception methods were eligible for inclusion in the study. However, those women who had been diagnosed with common mental illness, such as schizophrenia (cross-check their medical records when data collection) and women who were pregnant at the time of the study were excluded.

### Sampling technique and procedure

There were eight hospitals in Gamo Zone, namely Arba Minch comprehensive and teaching hospital, Arba Minch General Hospital, Gerese, Kamba, Chencha, Dilfana, Birbir, and Selam-Ber Primary Hospital. All these hospitals were included in this study and Study participants were selected consecutively, meaning that all eligible clients who visited the hospitals during a two-month study period were approached and interviewed. Accordingly, from Arba Minch General Hospital (221), Gersthe Primary Hospital (142), Chencha Primary Hospital (95), Selam Ber (110), Kamba primary hospital (91), Dilfana hospital (138), Birbir hospital (92), Arba Minch teaching hospital (113) were sampled and totally 1002 participants interviewed.

### Study variables and measurements

#### Study variables

**Dependent variable**: Major depression among hormonal contraception users (Yes/No, Binary) **Depression:** A structured Patient Health Questionnaire-9 (PHQ-9) was used to measure depression among women using hormonal contraceptives. PHQ-9 is a nine-item tool that is directly based on the nine diagnostic criteria for major depressive disorder in the Diagnostic and Statistical Manual Fourth Edition (DSM-IV). For each of the nine items, there is a value set from 0 to 3, with a total range of 0–27 response scale. The values are; (0 = not at all, 1 = several days, 2 = more than half of the days, 3 = nearly every day). By adding these values; the status of the study participants was determined as depressed if the PHQ-9 score is ≥ 10^5^.

**Independent variables**: Independent variable of this study has two sections: **socio-demographic characteristics:** Age, occupation, maternal education, smoking, marital status and residence. **Maternal and contraception use related factors:** type of contraception, duration of contraception use, history of abortion, number of children, last delivery, history of depression, and reason for intake and, history of depression, psychogenic medication uses.

### Data collection tool and procedure

An interviewer-administered and pretested questionnaires for socio-demographic and maternal and contraception factors were prepared by modifying some available literature reviews^19–24^. However, the PHQ-9, a standardized tool for assessing the severity of depression, was used without any modifications^5^. The tool consists of three sections: socio-demographic, maternal and contraception use factors and depression assessment part (PHQ-9). The questionnaire is designed in English and then translated into the local Amharic language. Based on their prior experience and command of the local (Amharic) language, 12 midwives were chosen to serve as data collectors, and eight master midwives supervised the overall data collection process and managed the data collection procedure. Data was collected using the ODK mobile application by six midwives.

### Data quality management

To ensure the data quality, a data collection tool was prepared after an intensive review of relevant literature and similar studies on socio-demographic and maternal and contraception factors. A properly designed data collection instrument was provided after training has been provided for data collectors and supervisors for two days. Pre-testing of the questionnaire was conducted of 50 participants two weeks before the commencement of the actual data collection at Sawula Primary Hospital from the nearest province to Gamo zone and all the necessary corrections were made based on the pretest result to avoid any confusion and for better completion of the questions. Every day the collected data was reviewed and cross-checked for completeness and relevance.

### Ethical consideration

This study was conducted in accordance with the ethical principles of the Declaration of Helsinki. Ethical approval for the original data collection was obtained from the Institutional Review Board (IRB) of Arba Minch College of Health Sciences (Protocol No. AMHCS/004/2025). Permission was also secured from the Gamo Zone Health Bureau and the respective public health facilities. The present study is a secondary analysis of previously collected data. Written informed consent was obtained from all participants during the original data collection process. For minors and participants unable to read or write, informed consent was obtained from legally authorized guardians as approved by the IRB.

Participants were informed about the purpose of the study, procedures, potential benefits and risks, and their right to refuse or withdraw at any time without consequence. No personal identifiers were recorded, and all data were anonymized prior to analysis to ensure confidentiality. All procedures complied with applicable national and institutional guidelines for research involving human participants.

### Preprocessing of data

A high-quality dataset is essential for machine learning to generate accurate predictions. To ensure unbiased model evaluation and avoid data leakage, all preprocessing steps were performed after the initial 80/20 train‑test split and within each fold of the fivefold cross‑validation. Missing values were handled using median imputation for numerical variables and mode imputation for categorical variables. Variables with many categorical levels, such as age, PHQ-9 score and duration of usage underwent discretization through binning methods to convert them into discrete/categorical values. Categorical variables were converted into numerical representations using one-hot encoding and label encoding, as appropriate. The imputation parameters and encoding schemes were derived solely from the training data and then applied to the test dataset. Crucially, the encoding schemes (such as label mappings and dummy variable categories) were applied to the test set and validation fold after being learnt solely from the training data. This maintains the integrity of performance evaluation by guaranteeing that no information from the test set or validation data affects the preparation of the training data. During cross-validation, the same systematic approach was followed: preprocessing (encoding and imputation) was only applied to the training section of each fold before being transferred to the associated validation fold^25^.

To prevent data leakage, SMOTE was applied only to the training portion of each fold during cross‑validation, and to the entire training set (after all preprocessing steps) before final evaluation on the hold‑out test set to assess model robustness and generalizability. The training dataset was partitioned into five subsets (folds); in each iteration, four folds were used for training and one fold was used for validation. This process was repeated five times so that each fold served once as the validation set. The area under the receiver operating characteristic curve (AUC-ROC) was calculated to assess the performance of the trained models. This approach ensured that no information from the test or validation data influenced model training, thereby preserving the validity of performance estimates.

### Hyperparameter tuning

Each machine-learning model’s performance was optimized through hyperparameter tuning. A predetermined grid of hyperparameter values was established for each algorithm, and fivefold cross-validation on the training dataset was used to determine the ideal parameters. The area under the receiver operating characteristic curve (AUC) was used as the main optimization metric to assess the model’s performance during this phase. The final model configuration was determined by combining the hyperparameters that produced the highest average AUC throughout the cross-validation folds. To avoid information leakage, all hyperparameter tweaking processes were carried out just within the training data. The performance of the optimized models’ generalization was then assessed using the independent test dataset. AUC was chosen as the primary optimization metric because it evaluates model performance across all classification thresholds, making it robust to class imbalance. Unlike accuracy or F1-score, AUC does not require selecting a specific probability cutoff, which is particularly important when comparing models on an imbalanced dataset.

### Data analysis tool

Descriptive statistics were employed in this study to use percentages and frequencies to characterize the socio-demographic parameters. Pre-processing the data, feature selection, data splitting, resolving unbalanced data, model construction, and model performance testing were among the phases of data analysis. The software utilized in this investigation was R 4.3 and Rstudio Window Version 1.12.

### Data split

For machine-learning approaches, the dataset was randomly divided into two parts: 80% of the dataset were used to train the model, while 20% were used to evaluate model performance. In order to predict major depression, a dataset consisting of 1002 instances or observations was utilized. Among these observations, 802 (80% of the total) were allocated for training the model, while the remaining 200 observations (20% of the total) were reserved for testing and evaluating the model’s performance. In this study, several suitable machine-learning algorithms, including Naïve Bayes, PART, logistic regression, decision tree, random forest, and XGBoost, were employed to predict major depression among reproductive-age women using hormonal contraceptives in southern Ethiopia. The training data was exposed to the SMOTE method, which creates synthetic samples using a K-nearest neighbors (KNN) algorithm^26^.

### Features of knowledge flow

**Fig. 1:**
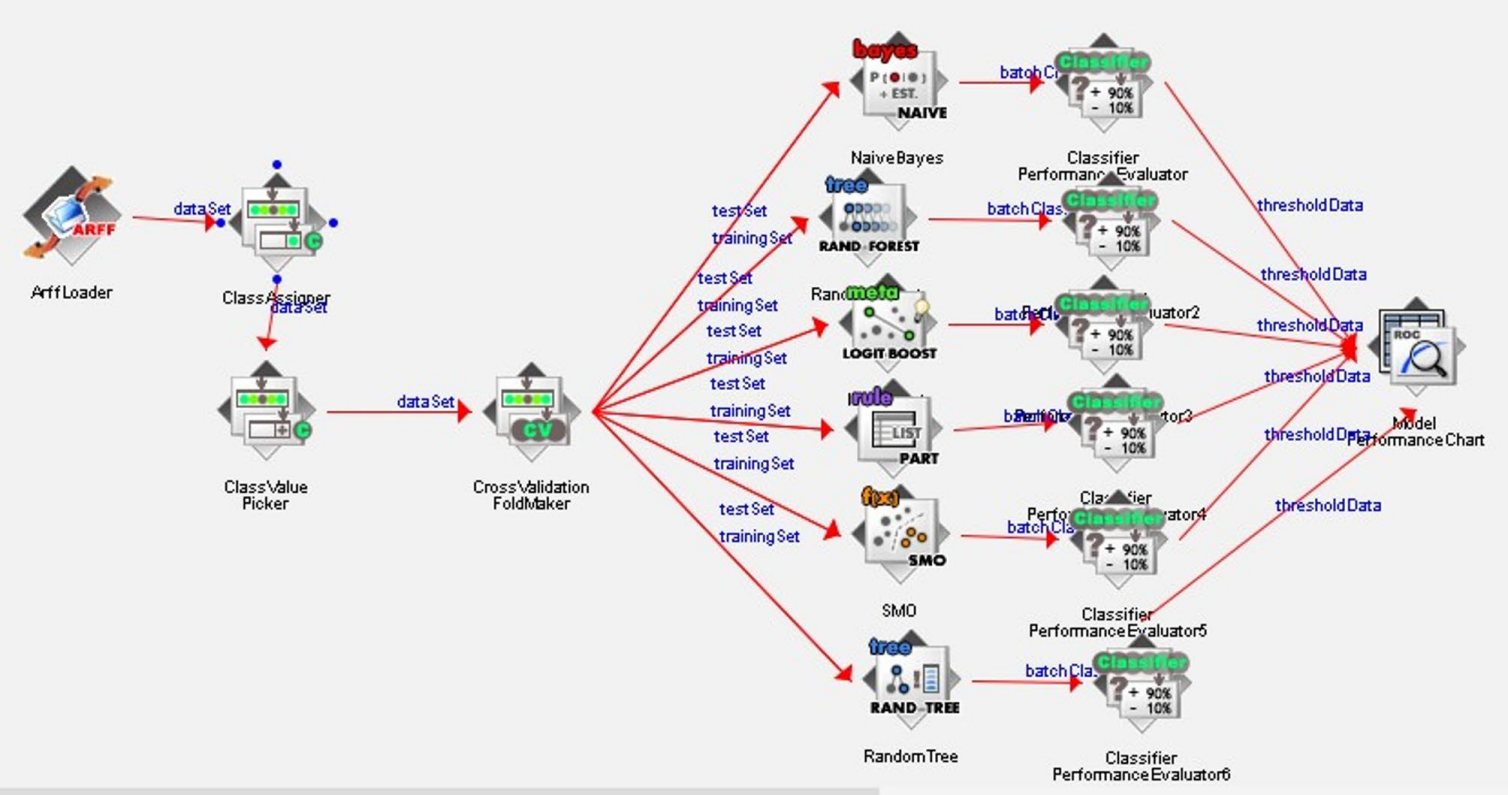
Features of knowledge flow of the included algorithms.

The knowledge flow feature in Weka software provides a user interface inspired by data-flow concepts for data processing and analysis. It allows for the handling of data in either incremental or batch mode. Knowledge flow supports the dissemination of insights and knowledge gained from trained models to inform decision-making processes. It encompasses various techniques such as data preprocessing, feature extraction, model training, evaluation, and deployment, ensuring a seamless flow of information throughout the machine learning pipeline using fold cross-validation^27^ (Fig. 1).

### Model evaluation

In this study, to build a predictive model for predicting major depression among hormonal contraception users in southern Ethiopia, various machine-learning algorithms such as Decision Tree, Random Forest, PART, logit model, Naive Bayes and XGBoost were employed. Based on the sample size (N = 1002) and the data has mixed categorical numerical, unbalanced structure, six algorithms were chosen. Because sample size directly affects how well a model can learn patterns without overfitting or under fitting, it is crucial when choosing a model. Because they require fewer parameters and have a lesser chance of overfitting, simpler models like Logistic Regression or Naive Bayes frequently perform better with smaller to moderate datasets (say N≈1000). On the other hand, intricate models with a large number of parameters might learn noise instead of broadly applicable patterns. However, because they reduce variance through strategies like bagging and boosting, ensemble approaches like Random Forest and XGBoost can still function well even with sparse data, though they still need to be carefully adjusted. Rule-based models (Decision Tree, PART) are also suitable because they handle mixed data types well and provide interpretability, but single trees can over fit if not constrained^28^.

In general, as sample size increases, more complex models become safer and more effective. With smaller datasets, the best practice is to balance model complexity with interpretability and regularization to ensure stable and generalizable performance.

The As shown in (Table 1), confusion matrix, the most used machine learning evaluation method, has been used to assess the performance of all the included algorithm^29,30^. In this study, the researchers used several metrics, including precision, sensitivity, specificity, F1-score, and AUC-ROC, to assess the performance of the model. They compared the predicted and actual classifications of major depression by analyzing the elements of the confusion matrix, such as true positive, false positive (FP), true negative, and false negative.

**Table 1:** Contingency table for confusion matrix.

	Predictive positive	Predictive negative
Actual positive	True positive (TP)	False negative (FN)
Actual negative	False positive (FP)	True negative (TN)

This study also employed the receiver operating characteristic (ROC) curve to evaluate the model’s performance by examining the relationship between sensitivity and specificity. Since the ROC curve is based on probabilities, the area under the curve (AUC) is an important measure of how well the model can distinguish between different classes. Typically, an AUC value above 80% is considered good, between 70 and 80% is considered fair, between 60 and 70% is considered poor, and below 60% is considered a failed performance^31^.

1 $$\mathrm{A}\mathrm{c}\mathrm{c}\mathrm{u}\mathrm{r}\mathrm{a}\mathrm{c}\mathrm{y} = \frac{\mathrm{T}\mathrm{P}+\mathrm{T}\mathrm{N}}{TP+TN+FN+FP}$$

2 $$\mathrm{P}\mathrm{r}\mathrm{e}\mathrm{c}\mathrm{i}\mathrm{s}\mathrm{i}\mathrm{o}\mathrm{n} =\frac{TP}{TP+FP}$$

3 $$\mathrm{R}\mathrm{e}\mathrm{c}\mathrm{a}\mathrm{l}\mathrm{l} = \frac{TP}{TP+FN}$$

4 $$\mathrm{F}1-\mathrm{s}\mathrm{c}\mathrm{o}\mathrm{r}\mathrm{e} = 2* \frac{Recall*Precision}{Recall+Precision}$$

## Results

### Socio-demographic characteristics of participants

A total of 1002 respondents were actually interviewed and provided accurate information in this study. The mean age of the women was 30.4 years (SD ± 6) with a minimum age of 18 and a maximum of 49 years, respectively. Respondents’ age was categorized based on classifications used in previously published peer-reviewed studies. More than half of the participants, 566 (56.5%), were within the 24–34 years age group. Additionally, the majority of respondents, 760 (75.8%), resided in urban areas. Nearly one-third, 293 (29.24%), had attained college-level education or higher. Half of the respondents, 560 (50.5%), were housewives (Table 2).

**Table 2:** Socio-demographic characteristics of study participants in Gamo zone, southern Ethiopia, 2026 (N = 1002).

Variables	Response	Frequency	Percent (%)
Age category	18–24	163	16.26
	25–34	566	56.50
	≥ 35	273	27.24
Residence	Rural	242	24.15
	Urban	760	75.84
	Divorced	16	2.66
	Widowed	7	1.16
	In cohabitation	568	94.35
	Separated	2	0.33
	Single	9	1.50
Maternal education	No formal education	173	17.26
	Primary (1–8)	259	25.84
	Secondary (9–12)	277	27.64
	College and above	293	29.24
Maternal occupation	Farmer	61	6.08
	Employed	435	43.41
	Housewife	506	50.50
Marital status	Divorced	42	4.19
	In cohabitation	945	94.31
	Separated	15	1.50

### Maternal and contraceptive factors

**Fig. 2:**
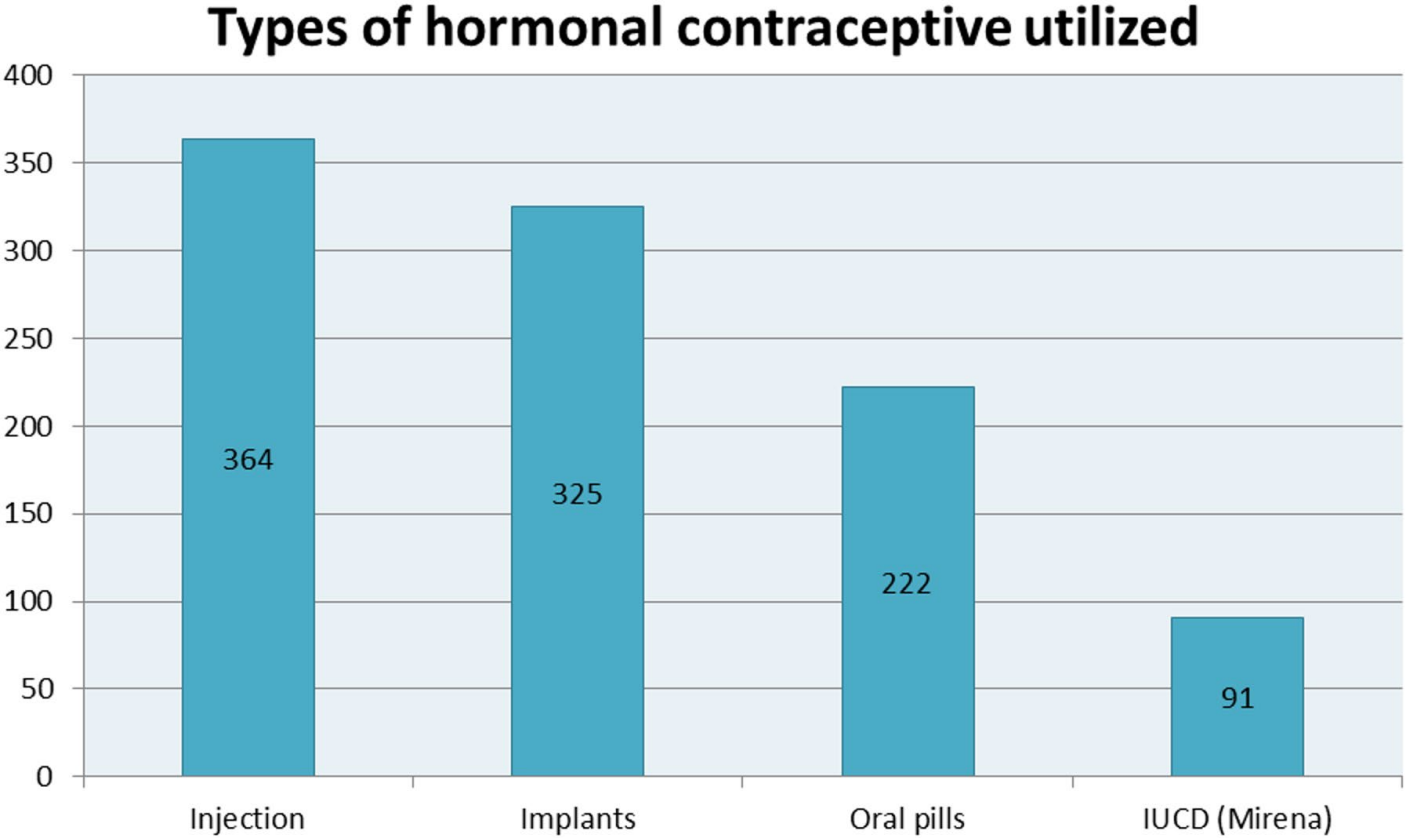
Types of contraceptives utilized by study participants in Gamo zone, southern Ethiopia, 2026 (N = 1002).

Regarding to maternal obstetric history, about 20% of the respondents had at least one abortion in their lifetime. Regarding to the use of hormonal contraceptives (Fig. 2), 364 (36.3%) of the respondents had ever used injectable contraceptives (Depo-Provera), while 325 (32.4%) had ever used implant. 452 (45%) of these users had been using the method for at least a year, and others had been using it for three years. Additionally, 94.4% of respondents used hormonal contraception for family planning, whereas 5.6% used it for therapeutic purposes, primarily to treat issues related to the menstrual cycle (Table 3).

**Table 3:** Maternal and Contraceptive factors of reproductive age women in Gamo zone, southern Ethiopia, 2026 (N = 1002).

Variables	Response	Frequency	Percent (%)
History of abortion	No	800	79.9
	Yes	202	20.1
Purpose of taking contraception	For Contraception	946	94.4
	Menstrual Disorder	56	7.58
Duration of usage	1–3 months	76	6.8
	3–6 months	115	11.47
	6–12 months	239	23.85
	1–3 years	452	45.10
	More than 3 years	120	12.00
Type of contraceptive	Oral pills	222	22.15
	IUCD(Mirena)	91	9.08
	Injection	364	36.32
	Implants	325	32.43
Ant-psychogenic drug	No	978	97.60
	Yes	24	2.40
History of psychiatric disorder	No	982	98.00
	Yes	20	2.00

### Prevalence of major depression southern Ethiopia

The Patient Health Questionnaire-9 (PHQ-9) was used to evaluate the study participants’ depression level. The instrument consists of a total possible score ranging from 0 to 27. The mean score in this study was 3.5 (SD ± 4.2), with observed scores ranging from 0 to 23. Based on standard PHQ-9 severity classification, 19.6% of respondents had mild depression (score 5–9), 12.6% had moderate depression (10–14), 0.33% had moderately severe depression (15–19), and 0.33% had severe depression (20–27). Major depression was defined as having a PHQ-9 cutoff score of ≥ 10, which has been demonstrated to have an 88% sensitivity and 88% specificity for diagnosing major depressive disorder^5^. As a result, 139 (13.87%) of the women has shown a major depression.

### Model evaluation and performance comparisons of ML algorithms to predict major depression

Six machine-learning algorithms were employed for predicting major depression among reproductive age women receiving hormonal contraception in Ethiopia. These algorithms consisted of PART, Naïve Bayes, random forest, decision tree, logistic regression, and XGBoost. To assess the performance of these algorithms, various evaluation metrics such as accuracy, precision, F1 score, and AUR (Area under Curve) were utilized. The predictive performance of the six machine learning models yielded Cohen’s Kappa values ranging from 0.185 to 0.480. While these values indicate slight to moderate agreement beyond chance, they should be interpreted alongside the high AUC (0.831) and F1-score (91%) achieved by the Random Forest model, which suggest strong overall classification reliability in a clinical context.

With a better balance between sensitivity and overall precision, the random forest model proved to be the most reliable classifier across all evaluation parameters. The model obtained a high F1-score (91%) and the highest AUC (0.831) on the test data, demonstrating outstanding discriminatory power, by using fivefold cross-validation and SMOTE to handle data imbalance. When moving from training to independent testing, the random forest maintained consistent accuracy (84.4%) and precision (93.2%), in contrast to other models that shown notable performance decay or over-fitting, particularly XGBoost. Together, these findings imply that the Random Forest ensemble strategy, which offers both high hit rates and low false-alarm rates, is the most dependable technique for detecting serious depression in this clinical setting.

Because of their excellent AUC scores, the PART Rules and XGBoost models were shown to be the second and third best performers, respectively, after the Random Forest. With an AUC of 0.820 and an F1-score of 89.4% on the test data, a little improvement over its cross-validation results PART Rules showed exceptional stability and generalization. With an AUC of 0.810, XGBoost trailed closely, retaining good precision (88.7%) and accuracy (84%) on the test set (Table 4).

**Table 4:** Performance metrics of the classifiers based on confusion matrix.

Machine learning models	Data set	Accuracy	AUC	Precision	Recall	F1-score
Logit model	5-Fold CV (%)	59.2	62.8	90	59	71
	Test data (%)	53	58.2	88.2	52.3	65.7
Random forest	5-Fold CV (%)	86	73.2	91.4	93	91
	Test data (%)	84.4	83.1	93.2	87.2	91
Decision tree	5-Fold CV (%)	76	67	88.6	75.3	86
	Test data (%)	73.3	67.3	89.5	78.5	83
PART	5-Fold CV (%)	80.8	71.4	70.6	88.7	89.4
	Test data (%)	82.4	82	82	86	89.4
Naive Bayes	5-Fold CV (%)	72	61	86	79	79
	Test data (%)	64.4	62	90	96	93
XGBoost	5-Fold CV (%)	84	73.5	86.4	93	90
	Test data (%)	84	81	88.7	85	84.8

Even with these advancements, care should be used when interpreting the findings. Several models showed high precision and recall values, however other models’ AUC values stayed moderate, indicating that predictive performance might not be reliable over various categorization thresholds. Performance measurements may also have been impacted by a possible class imbalance. Overall, ensemble approaches random forest in particular show encouraging predictive power, but the models have drawbacks that might limit their usefulness. Before considering clinical application, more feature refinement and validation using external datasets are required.

**Fig. 3:**
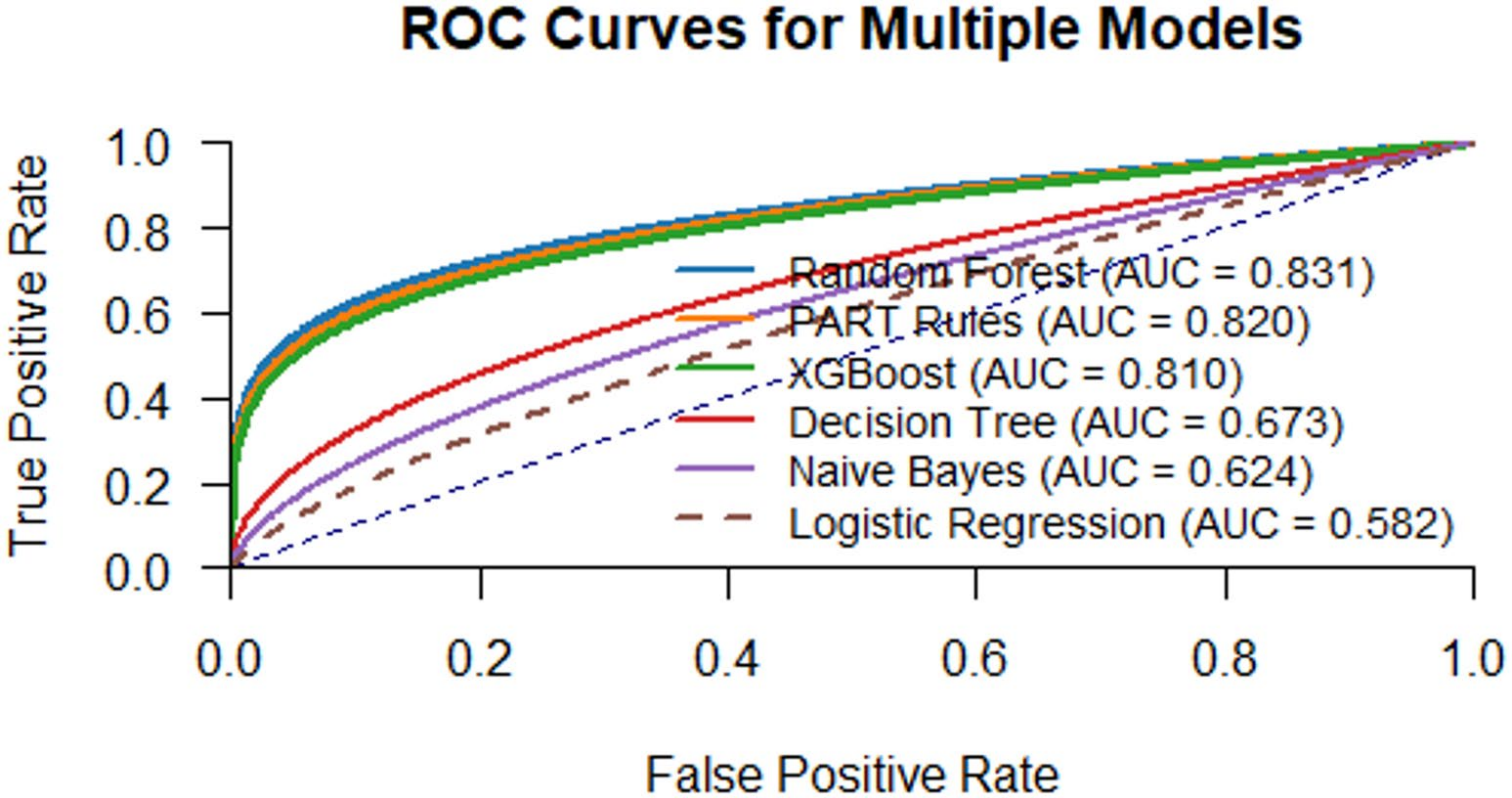
The ROC curve of the developed models.

The Receiver Operating Characteristic (ROC) curve in (Fig. 3) visually indicates the trade-off between the true positive rate and false positive rate for each classifier, with the Random Forest model emerging as the superior performer with an Area Under the Curve (AUC) of 0.831. To ensure the reliability of these results and address potential class imbalance within the dataset, a fivefold cross-validation (K = 5 CV) approach was employed alongside the application of the Synthetic Minority Over-sampling Technique (SMOTE). This combination ensures that the models were trained on balanced class distributions, preventing bias toward the majority class and providing a more robust estimate of performance across all folds. As a result, the curves for PART Rules (AUC = 0.820) and XGBoost (AUC = 0.810) show strong, stabilized predictive capabilities, while the Logistic Regression curve remains closest to the diagonal baseline, indicating it struggled to capture the underlying patterns even after balancing.

### Model interpretability

#### Random forest based feature importance

**Fig. 4:**
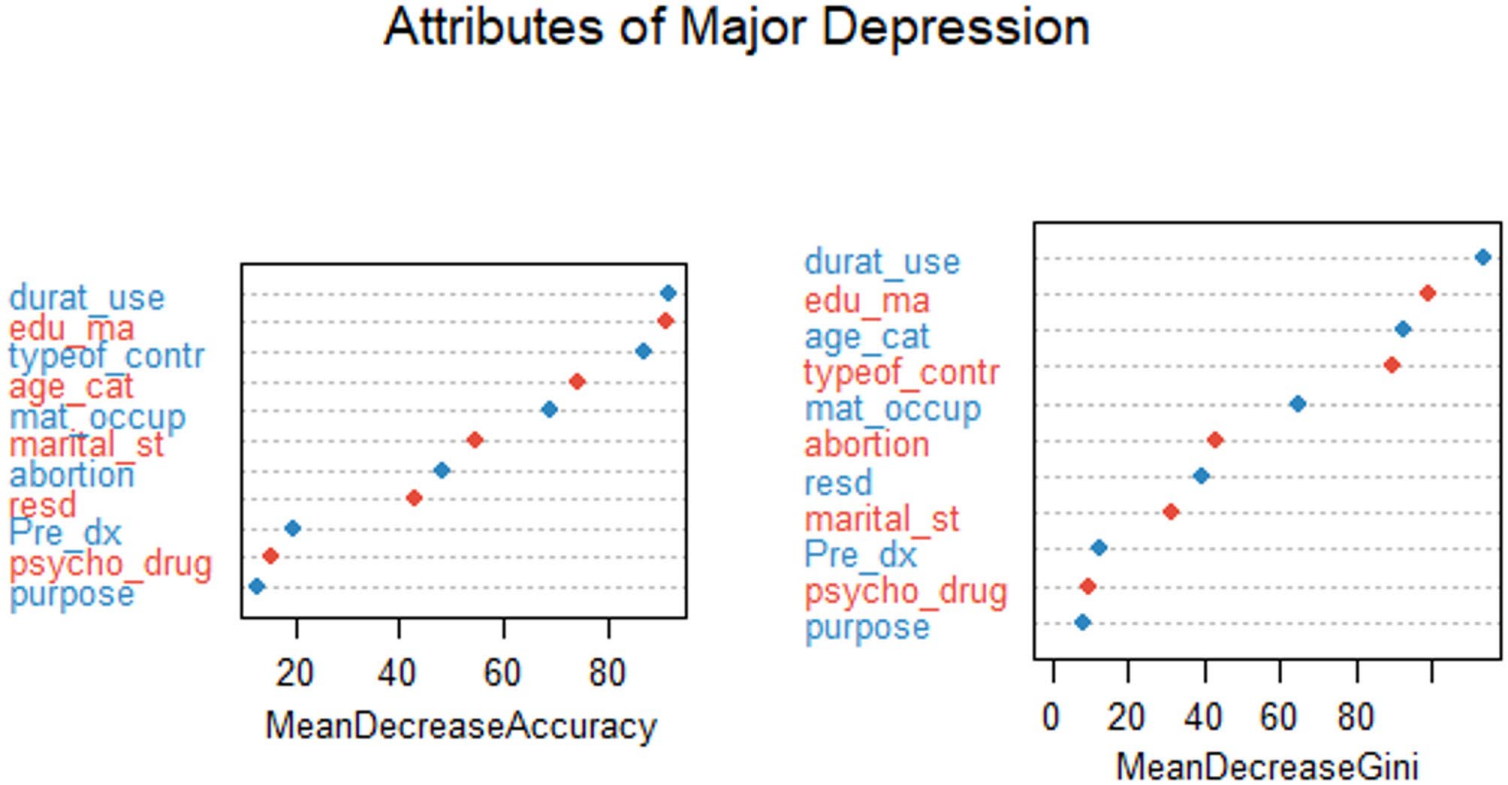
Random forest-based feature importance.

Important factors that influence developing of major depression was measured using Mean Decrease Accuracy and Mean Decrease Gini plot (as depicted in Fig. 4). The best performance model, random forest, was considered in order to choose important predictors. Accordingly, the most influential predictors of major depression among women using hormonal contraception were duration of contraceptive use, maternal educational level, and type of contraceptive method, age, occupation, marital status, and place of residence. In addition, a history of abortion, prior episodes of depression, and the use of anti-psychogenic medications were identified as important determinants of major depression, as illustrated in the figure. However, duration of usage, maternal education and type of contraception used are indeed the most significant variables, ranking highest in both Mean Decrease Accuracy and Mean Decrease Gini.

#### SHAP feature impact on model prediction

**Fig. 5:**
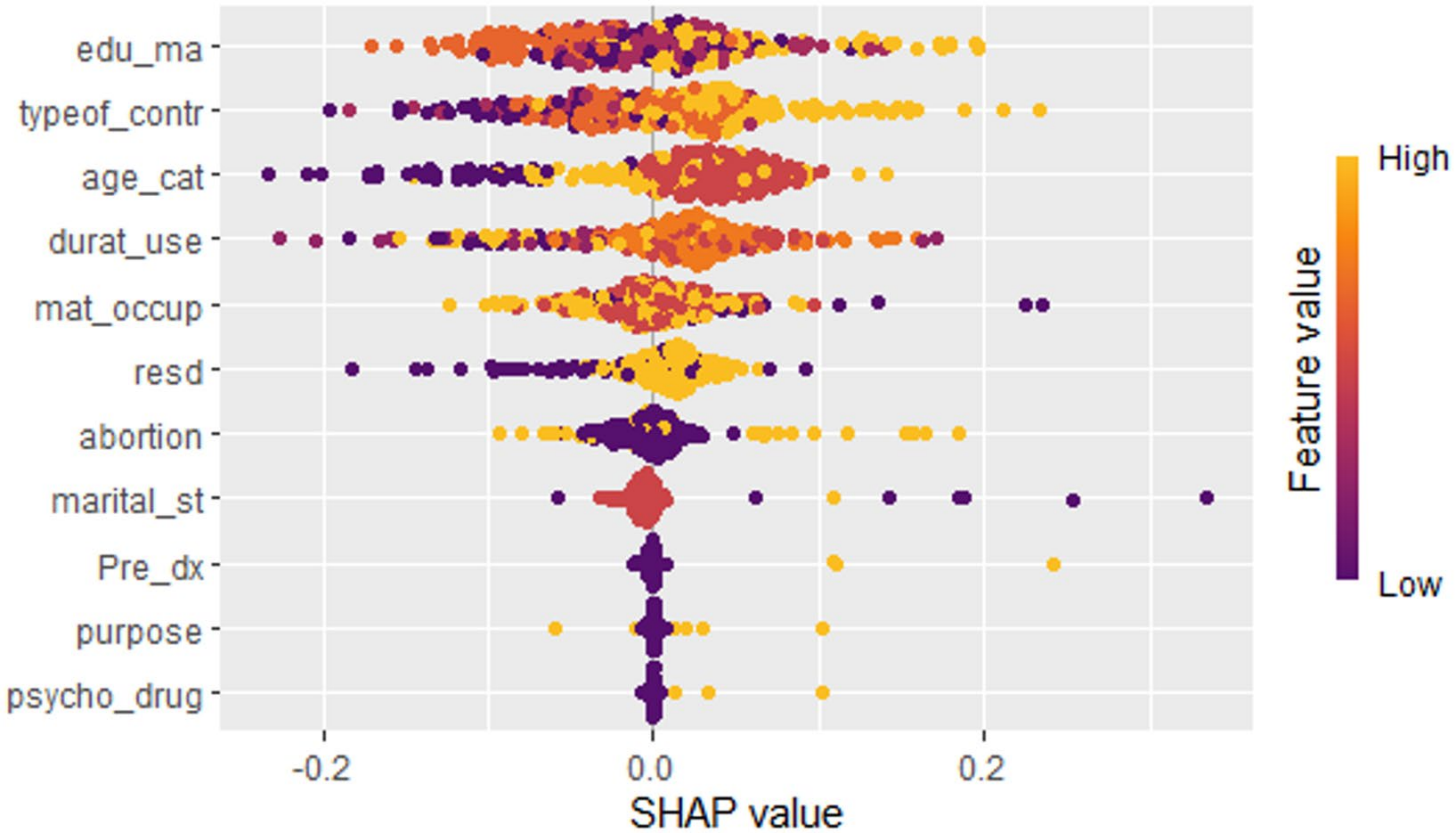
Presents a SHAP force plot that illustrates the impact of each feature on a model’s prediction using SHAP values.

The SHAP (SHapley Additive exPlanations) analysis presented in (Fig. 5) shows the feature importance scores for predicting a major depression. The features are listed on the vertical axis in descending order of their impact; the most important feature, type of contraception used (typeof_contr), is at the top. The horizontal axis represents SHAP values, where positive values (points to the right) indicate an increase in the predicted probability of major depression, while negative values (points to the left) indicate a decrease in the predicted probability. Positive values suggest that the feature increases the likelihood of the predicted outcome (developing major depression), while negative values indicate a decrease. Each point represents an individual observation, showing how a given feature contributes to moving the prediction toward or away from the outcome.

Features with many points on the left (negative SHAP values) are associated with predictions of lower probability of the outcome, while those on the right (positive SHAP values) are associated with predictions of higher probability.

It is crucial to remember that SHAP values do not indicate causal relationships; rather, they represent feature contributions to model predictions. As a result, rather than being important depression causes, the relationships found should be seen as predictive contributions. In contrast to Gini importance, the SHAP results show that residency, purpose, and method of contraception were the most important factors in the model’s prediction process, while age and abortion history contributed the least to the model’s output. The difference indicates that the type of contraceptive used and maternal education has a stronger and more consistent marginal contribution to the ultimate probability of a depression diagnosis across the entire model (SHAP), whereas duration of usage is very good at dividing the data into clean groups (Gini).

As shown in the Fig. 5, features on the left side of the SHAP force plot (shown in red) drive the model prediction in the direction of a higher probability of a major depression. On the other hand, characteristics on the right side (shown in brown) push the prediction in the direction of a reduced the probability of major depression. Each arrow’s length indicates the extent of that feature’s contribution by representing the magnitude of the accompanying SHAP value.

#### Waterfall and force plot interpretation

**Fig. 6:**
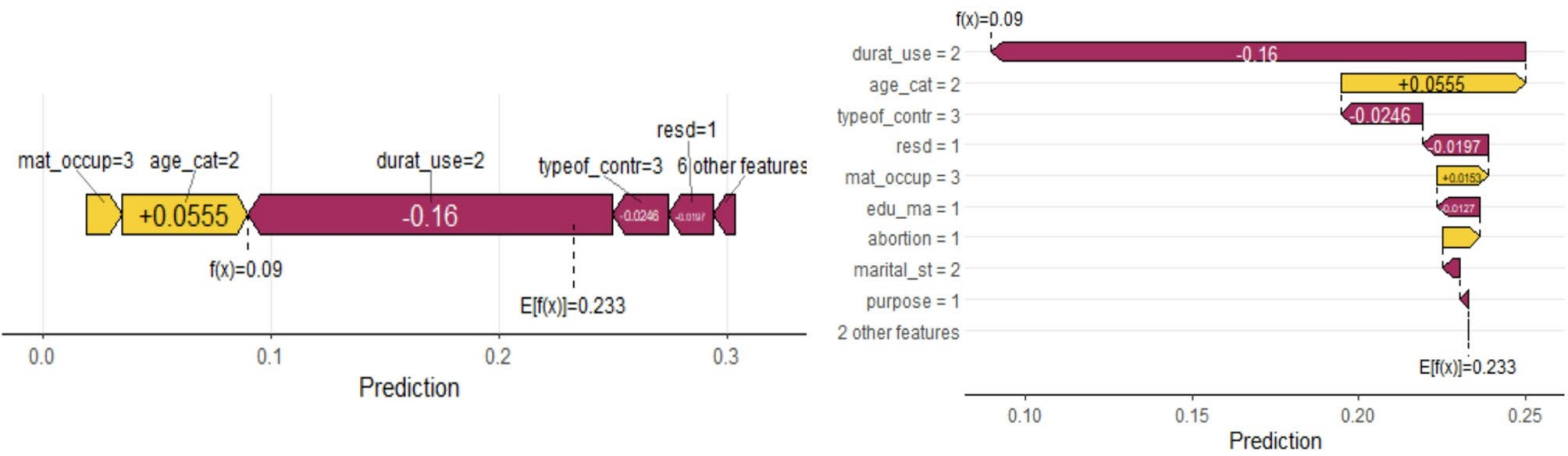
Shows both SHAP force plot and waterfall plot.

(As shown Fig. 6), the model’s prediction of a higher likelihood of major depression for positive cases was demonstrated using waterfall and force plots. The baseline forecast for a sample before taking into consideration the contributions of different variables is represented by the model’s expected output value on the x-axis (E[f(X)] = 0.233), which is where the plots start. The average prediction for the entire dataset is usually reflected in this baseline. Features that raise the projected probability are indicated by positive contributions (shown in brown); for instance, maternal occupation (being housewife) (maternal occupation = 3) and age between 18 and 24 increase the likelihood of major depression from 0.055 to 0.09.

Thus, The SHAP values for maternal occupation reveal that lower levels, such as being a housewife, have a positive contribution to the model’s prediction, which means that this variable is a risk factor for major depression. Similarly, the variable maternal age reveals that being younger than 34 years of age has a positive contribution to the predicted outcome, which means that younger age is a risk factor for depression. On the other hand, variables with dark red color have negative contributions, which decrease the predicted probability. The most significant protective variable is the duration of hormonal contraception use for more than three months (durat_use = 2), which decreases the predicted probability by − 0.16.

Figure 6 shows a SHAP force and waterfall plot that visualizes how each feature influences the model’s prediction. This plot highlights both the positive and negative contributions of features to the predicted outcome. Features that increase the predicted probability of developing major depression are shown on the left in brown, while features that decrease the probability are displayed on the right in dark red. The size of the arrows corresponds to the magnitude of the SHAP values, indicating the relative impact of each feature on the prediction.

### Association rule mining (exploratory analysis)

These exploratory findings were used to guide feature engineering and to generate hypotheses for future prospective studies. They are not intended as standalone clinical evidence. Association rule mining was used as an exploratory, unsupervised analysis in order to find co-occurring patterns among important clinical and demographic factors linked to major depression. After preprocessing, which included removing redundant features and converting pertinent variables into categorical factors, the Apriori method was used. To keep only correlations stronger than chance, rules were filtered using a lift value greater than one. After applying these criteria, five important association rules were discovered.

**Fig. 7:**
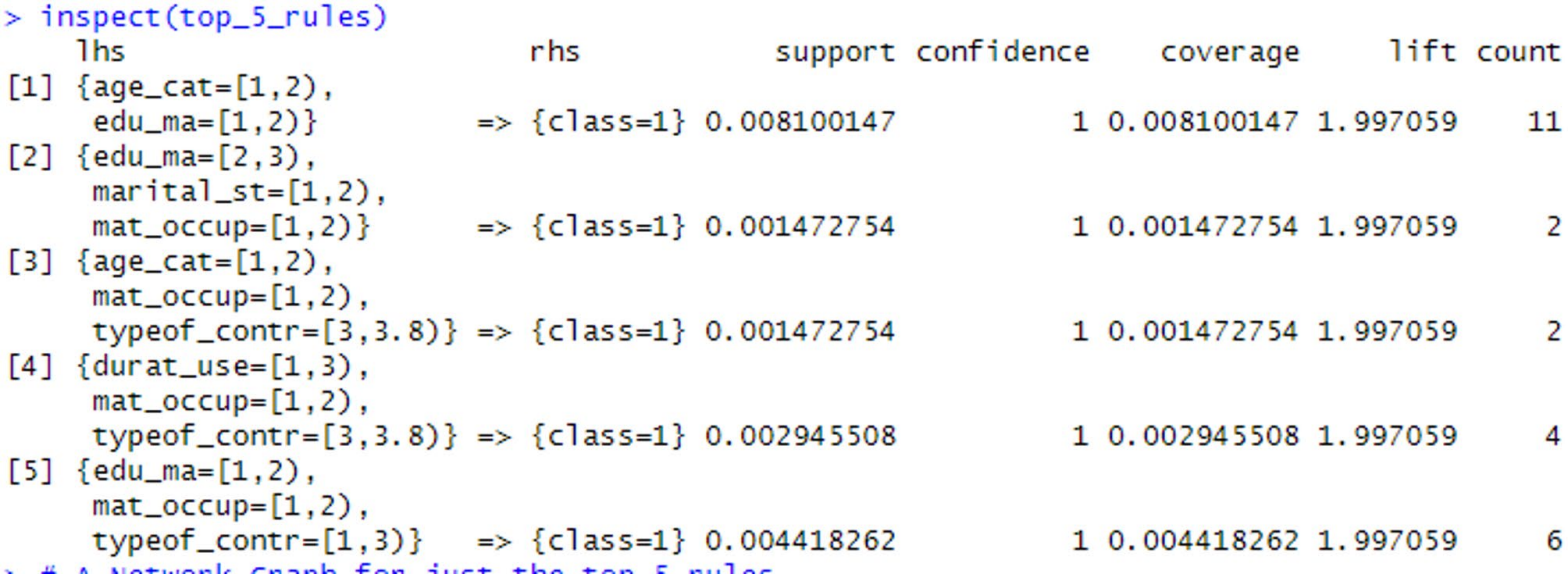
Association rule generation and knowledge extraction to predict a major depression.

Five association rules were identified, reflecting frequent co-occurrence patterns between variables such as age category, education level, marital status, occupation, and type/duration of hormonal contraceptive use. For example, some rules indicate that combinations of younger age groups with lower educational attainment, or specific occupational and contraceptive profiles, appear together more frequently among individuals classified as having depression in this dataset (Fig. 7).

**Rule 1**: (The Young/Education Profile): Among individuals with age in categories 1–2 (e.g., 18–34 years) and maternal education in categories 1–2 (e.g., no formal education), the model’s prediction of major depression occurred with 100% frequency in this subset (support = 11 cases). This pattern suggests an interaction between young age and low education in the dataset.

**Rule 4:** (Duration, occupation, and contraceptive type): The combination of how long someone has been using a hormonal contarception (durat_use) and their maternal occupation (mat_occup) creates a specific “vulnerability pocket”. When combined with specific types of hormonal contraception (typeof_contr), the lift is nearly 2.0, meaning this group is twice as likely to develop depression compared to the average.

**Fig. 8:**
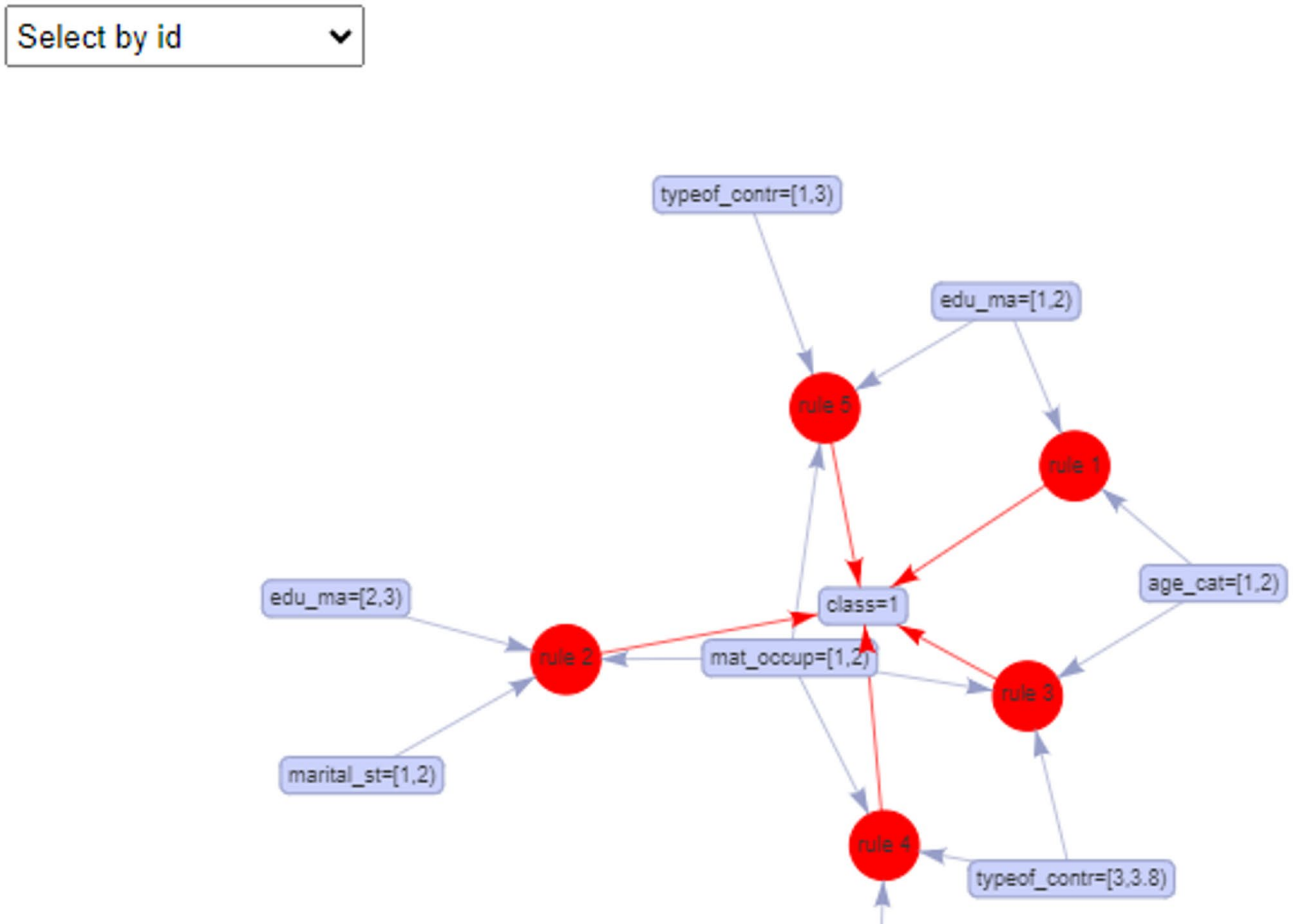
Important attributes selection, based on best performance algorithm to predict depression among study participants.

**Rule 5:** Maternal occupation (mat_occup) appears in rules 2, 3, 4, and 5, suggesting that this variable frequently co‑occurs with other features (education, contraceptive type) in patterns that lead to a high predicted probability of depression. This indicates that occupation may be an important interacting feature in the model’s predictions, not a causal mediator (Fig. 8).

## Discussion

This study provided a concise overview of algorithm comparisons for predicting major depression among reproductive-age women receiving hormonal contraceptives in southern Ethiopia using machine-learning algorithms. Six machine-learning algorithms, including Random Forest, Decision Tree, Naive Bayes, PART, XGBoost and Logistic Regression, were evaluated for their predictive capabilities in predicting major depression among women receiving hormonal contraceptives. Using the SMOTE (Synthetic Minority Over-sampling Technique) method, which addressed class imbalance and greatly increased accuracy and ROC (Receiver Operating Characteristic) scores, the dataset, was equalized to guarantee balanced predictions. Then, the prevalence of major depression was 13.87% before data balancing and after balancing the dataset using the SMOTE technique, this proportion was adjusted to 49.92%.

Recent advances in intelligent data-driven modeling have demonstrated the critical importance of designing architectures that remain robust under complex, real-world conditions. For instance, in civil infrastructure monitoring, Duan et al.^28^ introduced the DSYOLO model, a dynamic snake convolution-based segmentation framework for detecting tunnel lining cracks in challenging environments^28^. In the field of clinical prediction, similar challenges arise for example; predicting major depression using diverse patient populations involves managing class imbalance, non-linear feature interactions, and varying data quality. The present study builds on this broader methodological landscape by systematically evaluating six machine-learning models (Logistic Regression, Random Forest, Decision Tree, PART, Naive Bayes, XGBoost model) within a rigorous preprocessing and validation pipeline, with a particular focus on maintaining robust performance under real-world data conditions.

Thus, The Random Forest algorithm exhibited the highest performance metrics, achieving accuracy of 84.7%, precision of 42%, sensitivity of 58%, F1-score of 48%, and an AUC of 0.82. The RF model demonstrated superior prediction accuracy and AUC values compared to the other models assessed. This study represents a single investigation aimed at predicting the probability of developing major depression among hormonal contraception users. Given the limited number of comparable studies in this specific population, the absence of direct contrasts for discussion is understandable and should be considered when interpreting the findings.

o improve the interpretability of the machine-learning model SHAP was applied. Researchers can determine which characteristics have the greatest influence on outcomes by measuring each feature’s contribution to individual forecasts. This increases the data’s transparency and practical significance for healthcare decision-making. Accordingly, the SHAP findings highlight type of contraception, residence, and purpose as the most significant influencing factors, while age and history of abortion had the lowest impact on the model’s predictions for major depression.

The other objective of this study was to identify important characteristics that might be used to predict major depression. According to the insights derived from the SHAP analysis; maternal occupation and age are the most influential factors for predicting major depression among reproductive age women receiving hormonal contraception. Thus, maternal occupation that is being housewife is important feature of developing major depression. This finding is similar with finding from primary studies conducted in United State^32^ and Mogadishu, Somalia^19^. Another finding also support the finding stating that Maternal depression affects 10–15% of postpartum women, with housewives often facing higher risks due to isolation, lack of independence, and intense, 24/7 caregiving demands^33^. This importance of feature implies that if the women are completely housewife and using hormonal contraception the probability of developing a major depression is high.

Similarly, the variable maternal age reveals that being younger than 34 years of age has a positive contribution to the predicted outcome, which means that younger age is a risk factor for developing a major depression. This implies that if women are using hormonal contraception and younger than age of 34 years old they are highly predictive of developing a major depression. This finding is consistent with find from study in conducted in Jimma Town Public Health Facilities, Southwest Ethiopia^34^. This may be due to the amygdala, prefrontal cortex, and hippocampus, which are important regions of the brain related to emotions, are still maturing during this age and may be particularly sensitive to changes in sex hormones^35^.

While, the SHAP value indicates the most significant protective variable is the duration of hormonal contraception use for more than three months (durat_use = 2), which decreases the predicted probability by − 0.16. This implies if a woman uses hormonal contraception for more than three months the probability of developing a major depression is decrease by 16%. This finding is consistent with finding from study conducted in United State pointing the risk for depressive symptoms decreased as the duration of birth control pill use (DBCPU) increased^36^.

Furthermore, this study generates five association rules using the significant attributes derived from the top-performing machine-learning model (Random forest). Following the preprocessing of the dataset, including the conversion of relevant variables to categorical factors and the removal of constant and redundant attributes, association rule mining was performed using the Apriori algorithm. Accordingly, rule (1) suggests that limited educational attainment at a young age is a strong precursor for developing a major depression and the combination of how long someone has been using a hormonal contarception (durat_use) and their maternal occupation (mat_occup) creates a specific “vulnerability pocket” (rule 4) are clinically significant rules.

### Implication of the study

The machine-learning analysis highlights key factors influencing the risk of developing major depression among hormonal contraception users. Features such as younger maternal age, lower educational levels, and being a housewife were associated with higher predicted probabilities, suggesting these groups may be more vulnerable. Conversely, prolonged use of hormonal contraception emerged as a protective factor, reducing the likelihood of depression. These findings imply that targeted mental health monitoring and counseling could be prioritized for high-risk subgroups, while also informing clinicians and policymakers about potential protective behaviors or interventions that may mitigate depression risk in this population.

Furthermore, while Random Forest shows promising predictive performance, independent external validation on larger, multi-center datasets is essential before clinical translation. Future work should also explore feature selection to improve generalizability. Additionally, while some models attained comparatively high F1-scores and accuracy, the AUC values for a number of algorithms were only moderate, suggesting a limited capacity for discrimination. This implies that the performance of the model could not be constant across various classification levels. Secondly, the dataset’s class imbalance might have affected performance metrics, thereby increasing F1-score, accuracy, and precision while concealing subpar performance in properly recognizing occurrences of minority classes.

## Conclusion and recommendations

Six (6) machine-learning models such as Random Forest, Decision Tree, XGBoost, Naive Bayes, Logistic Regression, and PART were developed and evaluated for their ability to predict major depression among users of hormonal contraception. Random Forest performed the best overall (test AUC = 0.831, F1 = 0.91), although precision and recall show that the model’s positive predictive value is still low. Maternal occupation, younger age, and lower educational attainment were found to be the model’s best predictive factors by feature importance analysis. It is not appropriate to consider these findings as causal risk factors because they are exploratory. The model requires external validation on independent, multi‑center datasets before any consideration of clinical decision support. Future work should focus on feature refinement, prospective validation, and assessment of clinical utility in real‑world settings.

## Data Availability

The dataset used in the current study are not available to the general public. Since not all participants gave us permission to publish their data, the raw data are still available from corresponding author with justifiable request.

## Abbreviations

AUC: Area under curve
EDHS: Ethiopia demographic and health survey
COC: Combined oral contraception
HC: Hormonal contraception
OC: Oral contraception
IBD: Institutional Based Delivery
ROC: Receiver operating characteristic
PHQ-9: Patients health questionnaires
PART: Partial decision tree
WHO: World Health Organization
